# A systematic review and meta-analysis of balance training in patients with chronic ankle instability

**DOI:** 10.1186/s13643-024-02455-x

**Published:** 2024-02-12

**Authors:** Yiwei Guo, Tianyue Cheng, Zihao Yang, Yonglei Huang, Ming Li, Taoli Wang

**Affiliations:** 1 Department of Rehabilitation, Xinhua Hospital, School of Medicine, Shanghai Jiaotong University, Shanghai, 200092 China; 2 Shanghai Yangzhi Rehabilitation Hospital (Shanghai Sunshine Rehabilitation Center), Tongi University School of Medicine, Shanghai, China; 3 Department of Physical Therapy, Tongji University School of Medicine, Shanghai, China; 4 Jiading Town Street Community Health Service Center, Shanghai, China; 5 Sanlin Community Health Service Center, Shanghai, China

**Keywords:** Chronic ankle instability, Balance training, Rehabilitation, Care, Treatment, Meta-analysis

## Abstract

**Background:**

Chronic ankle instability (CAI) is a common yet serious problem for elder patients. This meta-analysis aimed to evaluate the effects of balance training for CAI, to provide evidence for the clinical treatment, and care of CAI patients.

**Methods:**

Two investigators searched PubMed, EMBASE, Science Direct, Web of Science, Cochrane Library, China National Knowledge Infrastructure, Wanfang, and Weipu Databases up to May 20, 2023, for randomized controlled trials (RCTs) on the effects of balance training for CAI. The mean difference (MD) with 95% confidence intervals (95%CIs) was calculated for each outcome with a fixed or random effect model. Review Manager 5.3 software was used for meta-analysis.

**Results:**

Nine RCTs involving 341 patients were included. Meta-analysis results showed that compared with blank controls, balanced training treatment of CAI could significantly improve the score of CAI [MD = 3.95, 95% CI (3.26, 4.64), *P* < 0.00001], SEBT-PM [MD = 4.94, 95% CI (1.88, 8.00), *P* = 0.002], SEBT-PL [MD = 5.19, 95% CI (1.57, 8.81), *P* = 0.005], and FAAM Sports [MD = 17.74, 95% CI (14.36, 21.11), *P* < 0.00001]. Compared with strength training, balance training treatment of CAI improved the score of CAIT [MD = 2.36, 95% CI (0.29, 4.44), *P* = 0.03], FAAM-ADL [MD = 4.06, 95% CI (1.30, 6.83), *P* = 0.004].

**Conclusion:**

The analysis outcomes indicate that balance training enhances daily activity capability, motor function, and dynamic balance to different extents. Additionally, when comparing the results of balance training and strength training, no significant difference was observed between the two methods in improving the dynamic stability of CAI patients. However, it is noteworthy that balance training exhibits a more pronounced impact on enhancing functional scale scores.

## Background

Ankle joint injury is an important medical care problem. Ankle sprains affect about 8% of the general population, and the recurrence rate is as high as 80% in patients engaging in high-risk sports [1, 2]. A previous study [3] has reported that 40%-55% of patients still have residual symptoms of ankle joint six months after the occurrence of ankle sprain. Lateral ankle instability refers to the injury of the lateral ligament caused by excessive supination of the ankle or varus of the hind foot and the continued development of residual symptoms [4]. Chronic ankle instability (CAI) refers to the instability of the lateral ankle joint caused by repeated ankle sprains. Patients with CAI may have obstacles in proprioception, neuromuscular control, strength, and posture control alone or simultaneously [5, 6].

For CAI patients, conservative treatment is the first choice. After active conservative treatment, if the symptoms still do not improve, surgical treatment can be attempted [7]. The surgical treatment is usually the repair or reconstruction of ankle ligaments, for the purpose of strengthening the stability of the ankle joint and avoiding sprain again [8]. Postoperative rehabilitation methods for CAI include physical therapy, strength training, joint range of motion training, balance training, proprioception training, gait training, etc. Previous studies [9, 10] have shown that early and standardized rehabilitation after CAI can avoid and improve ankle joint range of motion limitation, proprioception, muscle strength, balance ability decline, gait abnormalities, and other dysfunctions.

Balance training refers to rehabilitation training aimed at restoring or improving body balance ability. It includes static balance training, dynamic balance training, reactive balance training, sensory integration balance training, and functional activity balance training [11]. The Cumberland Ankle Instability Tool (CAIT), Foot and Ankle Ability Measure ADL (FAAM-ADL), Foot and Ankle Ability Measure Sports (FAAM Ports), and Star Deviation Balance Test (SEBT) are commonly used tools for the evaluation of the effects of balance training. Previous studies [12, 13] have shown the effectiveness of balance training for sensory-motor and functional activities of CAI patients in terms of function, stability, strength, joint range of motion, balance, and other aspects. However, some studies [14, 15] have shown that balance training is not superior to other conservative treatments in terms of self-reported function, ankle strength, balance ability, and range of motion of CAI patients. The effectiveness of balance training on functional recovery of CAI patients is still controversial among the findings of related studies, and the related reports focused on the role of balance training in CAI patients are few. Therefore, the purpose of this systematic review and meta-analysis is to comprehensively and quantitatively analyze the available evidence and compare the role of balance training in self-reported function and dynamic balance stability, to provide reliable evidence for the clinical treatment of CAI.

## Methods

The Preferred Reporting Items for Systematic Reviews and Meta-Analyses (PRISMA) methodological review framework was used in this systematic review and meta-analysis to ensure systematic data collection and analysis of literature [16].

### Inclusion and exclusion criteria

The inclusion criteria were as follows: the study design was a randomized controlled trial (RCT). The study population was patients with chronic ankle instability (CAI), including functional ankle instability (FAI) and mechanical ankle instability (MAI). The experimental group used balance training as the intervention method. The balance training included static balance training, dynamic balance training, reactive balance training, sensory integration balance training, and functional activity balance training. The control group used unbalanced training as the intervention method, including no intervention and strength training. The outcome indicators shall at least include one of the CAIT, FAAM-ADL, FAAM Ports, and SEBT. This meta-analysis excluded the repetitively reported literature and the studies reported in languages other than Chinese and English.

### Search strategy

Two investigators searched PubMed, EMBASE, Science Direct, Web of Science, Cochrane Library, China National Knowledge Infrastructure, Wanfang, and Weipu Databases. The two investigators accessed the databases simultaneously. The retrieval time limit was between the establishment of the database and May 20, 2023. The Medical Subject Headings (MeSH) terms used for the literature search were as follows: (“ankle instability” OR “recurrent ankle sprain” OR “chronic ankle instability” OR “chronic lateral ankle instability” OR “CAI” OR “CLAI” OR “functional ankle instability") AND (“balance”) AND (“rehabilitation” OR “physical therapy” OR “health management” OR “physiotherapy” OR “exercise” OR “training”). We adapted the search strategy in different databases to retrieve the relevant studies. The references in the reference lists of potentially included reports were screened to identify additional articles that might meet the inclusion criteria.

### Literature screening and data extraction

According to the title and abstract of the literature, the two researchers independently completed the preliminary screening of the literature. After removing the duplicate literature, they searched and read the original text, and screened the literature that met the inclusion criteria for the second time. Any disagreements between reviewers during the screening process were submitted to a third reviewer and discussed collectively. The data extracted in this meta-analysis included the name of the first author, publication time, sample size, age, duration, frequency, and intensity of the intervention and the related outcomes such as CAIT, FAAM-ADL, FAAM Ports, and SEBT.

### Quality evaluation

Two researchers independently evaluated the methodologic quality using the Physiotherapy Evidence Database (PEDro) scale [17]. If agreement in an RCT’s score was not achieved, the authors discussed and came to a consensus on a score. The choice of the PEDro scale over other bias assessment tools recommended for RCTs was deliberate. The PEDro scale is widely recognized and specifically designed for the quality evaluation of RCTs related to physiotherapy. We believed that the PEDro scale was more targeted and appropriate for our specific context.

### Statistical analysis

We used Review Manager 5.3 (RevMan 5.3) [18] for meta-analysis. Continuity variables were expressed by mean difference (MD) and 95% confidence interval (CI). A chi-square test was used to judge the difference among the included RCTs. When the *P* > 0.05, *I*^2^ < 50%, there was homogeneity in the results. When *P* ≤ 0.05 and *I*^2^ ≥ 50% indicated that the results were heterogeneous. A fixed effect model was used for homogeneous data, and the random effect model was used for heterogeneous data. Publication bias was assessed by funnel plots and the Egger regression test in this meta-analysis. Funnel plot, a simple scatter plot of the intervention effect estimates from each study, was plotted against some measure of each study’s size or precision. Ten or more studies were required for the significant evidence of funnel plot. Further, we conducted sensitivity analyses to identify the influence of a single study on the whole synthesized results. *P* < 0.05 indicated that the difference was statistically significant.

## Results

### Study inclusion

**Fig. 1:**
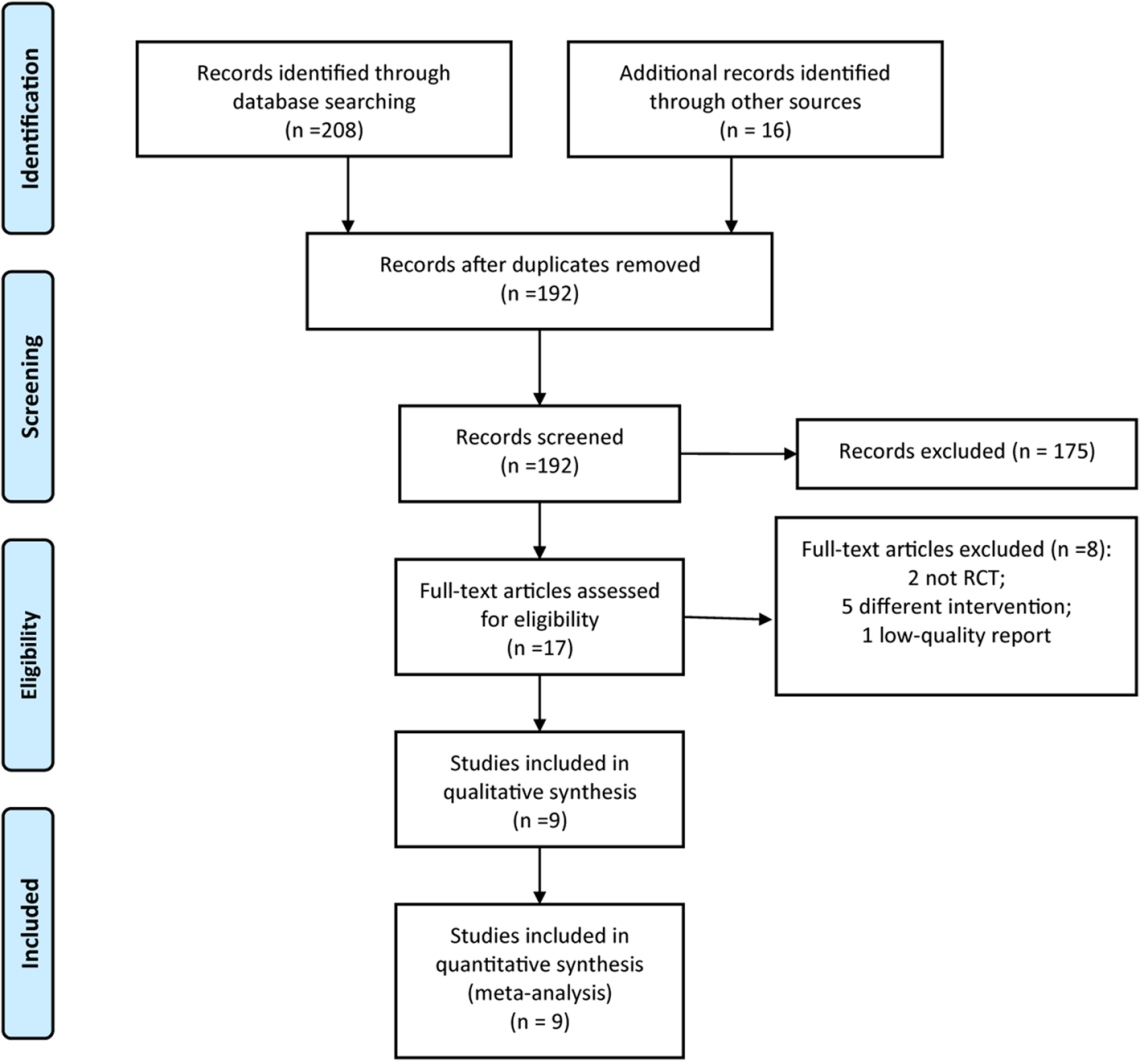
PRISMA flow diagram of RCT inclusion

A total of 224 reports were obtained from the initial database searches. After the removal of duplicates, 192 reports were screened, and 175 reports were excluded after the first screening of the title and abstract, thus 17 reports were further included for full-text screening. According to the inclusion and exclusion criteria, nine RCTs [19–27] were finally included in this meta-analysis. The PRISMA flowchart of study selection is presented in Fig. 1.

### Characteristics and quality of included RCTs

The characteristics of the included studies are presented in Table 1. Of the included RCTs, 341 patients were involved, and 165 patients underwent balance training. There are eight groups of “balance training versus no intervention” trials and two groups of “balance training versus strength training” trials. The outcome indicators of the study included seven groups of CAIT, three groups of FAAM, and seven groups of SEBT.

**Table 1:** The characteristics of included RCTs

RCT ID	Sample size		Age		Interventions		Outcomes
	Experimental group	Control group	Experimental group	Control group	Experimental group	Control group	
Kyung-Min 2021 [22]	25	23	29.76 ± 10.009	29.67 ± 9.407	Balance training, 20 min, 3 days/week, lasting for 6 weeks	Blank control	CAIT, FAAM, SEBT
Cain 2020 [19]	10	11	16.20 ± 1.14	16.45 ± 1.04	Balance training, 3 days/week, lasting for 4 weeks	Strength training, 3 days/week, lasting for 4 weeks	CAIT, FAAM, SEBT
Cruz-Diaz 2015 [21]	35	35	31.89 ± 10.52	28.83 ± 7.91	Balance training, lasting for 6 weeks	Blank control	CAIT, SEBT
Rafael 2018 [26]	16	17	21.8 ± 2.1	23.6 ± 3.4	Balance training, 20 min, 3 days/week, lasting for 6 weeks	Blank control	SEBT
Yi-Fen 2018 [25]	15	15	26.9 ± 5.8	27.9 ± 6.6	Balance training, 20 min, 2 days/week, lasting for 4 weeks	Blank control	CAIT
Cynthia 2017 [27]	20	20	22.60 ± 5.89	21.45 ± 3.24	Balance training, 20 min, 3 days/week, lasting for 4 weeks	Strength training, 3 days/week, lasting for 4 weeks	CAIT, FAAM, SEBT
Cain 2017 [20]	11	11	16.45 ± 0.93	16.55 ± 1.29	Balance training, 5 min, 3 day/week, lasting for 4 weeks	Blank control	SEBT
Shelley 2016 [24]	17	17	22.94 ± 2.77	23.18 ± 3.64	Balance training, 5 min, 3 days/week, lasting for 6 weeks	Blank control	SEBT
Liang 2015 [23]	16	16	34.31 ± 10.77	34.06 ± 11.49	Balance training, 15 min, 5 days/week, lasting for 4 weeks	Blank control	CAIT

*CAIT* Cumberland Ankle Instability Tool, *FAAM-ADL* Foot and Ankle Ability Measure ADL, *FAAM-Sports* Foot and Ankle Ability Measure Sports, *SEBT* Star Excursion Balance Test

The PEDro scores for the quality of the included studies are presented in Table 2. There were three RCTs with 7 points, five RCTs with 6 points, and one RCT with 5 points.

**Table 2:** The methodologic quality of included RCTs by Physiotherapy Evidence Database (PEDro) scale

RCT ID	1	2	3	4	5	6	7	8	9	10	11	Total
Kyung-Min 2021 [22]	Yes	Yes	Yes	Yes	No	No	No	Yes	Yes	Yes	Yes	7
Cain 2020 [19]	Yes	Yes	Yes	No	No	No	No	Yes	Yes	Yes	Yes	6
Cruz-Diaz 2015 [21]	Yes	Yes	Yes	No	No	No	No	Yes	Yes	Yes	Yes	6
Rafael 2018 [26]	Yes	Yes	Yes	Yes	Yes	No	No	No	Yes	Yes	Yes	6
Yi-Fen 2018 [25]	Yes	Yes	No	Yes	No	No	No	Yes	No	Yes	Yes	5
Cynthia 2017 [27]	Yes	Yes	Yes	Yes	No	No	No	Yes	No	Yes	Yes	6
Cain 2017 [20]	Yes	Yes	No	Yes	Yes	No	No	Yes	Yes	Yes	Yes	7
Shelley 2016 [24]	Yes	Yes	No	Yes	Yes	No	No	Yes	Yes	Yes	Yes	7
Liang 2015 [23]	Yes	Yes	No	Yes	No	No	No	Yes	Yes	Yes	Yes	6

**Fig. 2:**
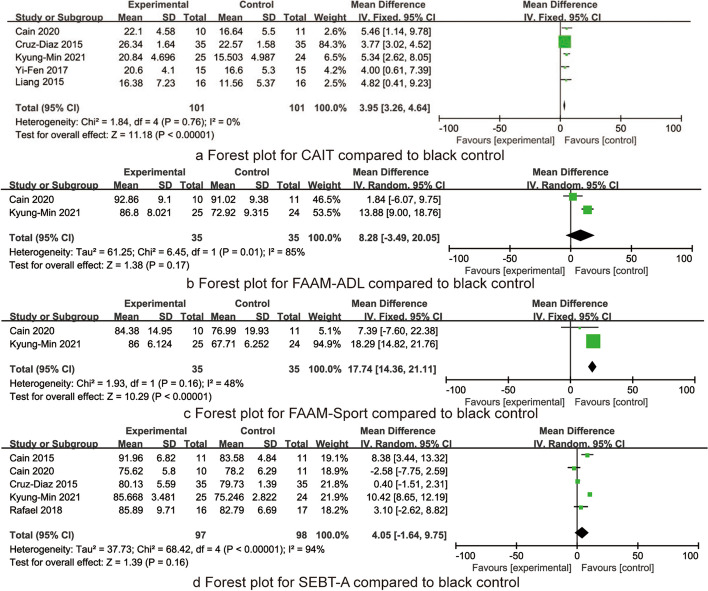
The forest plots for CAIT, FAAM-ADL, FAAM-Sports, and SEBT-A compared to black control

Five RCTs reported the CAIT after the intervention, including 202 patients. There was homogeneity (*I*^2^ = 0%, *P* = 0.76), and the fixed effect model was used. Meta-analysis results showed that compared with the blank control group (controls without interventions), the CAI score of the balance training group was significantly improved [MD = 3.95, 95% CI (3.26, 4.64), *P* < 0.001, Fig. 2a].

Two RCTs reported the FAAM-ADL after the intervention, including 70 patients. There was heterogeneity (*I*^2^ = 85%, *P* = 0.001), and the random effect model was used. Meta-analysis results showed that there was no significant difference in the FAAM-ADL score between the balance training group and the blank control group [MD = 8.28, 95% CI(− 3.49, 20.05), *P* = 0.17, Fig. 2b].

Two RCTs reported the FAAM-Sports after the intervention, including 70 patients. There was homogeneity (*I*^2^ = 48%, *P* = 0.16), and the fixed effect model was used. Meta-analysis results showed that compared with the blank control group, the FAAM- Sports score of the balance training group was significantly improved [MD = 17.74, 95% CI(14.36, 21.11), *P* < 0.001, Fig. 2c].

Five RCTs reported the SEBT-A after the intervention, including 195 patients. There was heterogeneity (*I*^2^ = 94%, *P* < 0.001), and the random effect model was used. Meta-analysis results showed that there was no significant difference in the SEBT-A score between the balance training group and the blank control group [MD = 4.05, 95% CI (− 1.64, 9.75), *P* = 0.16, Fig. 2d].

**Fig. 3:**
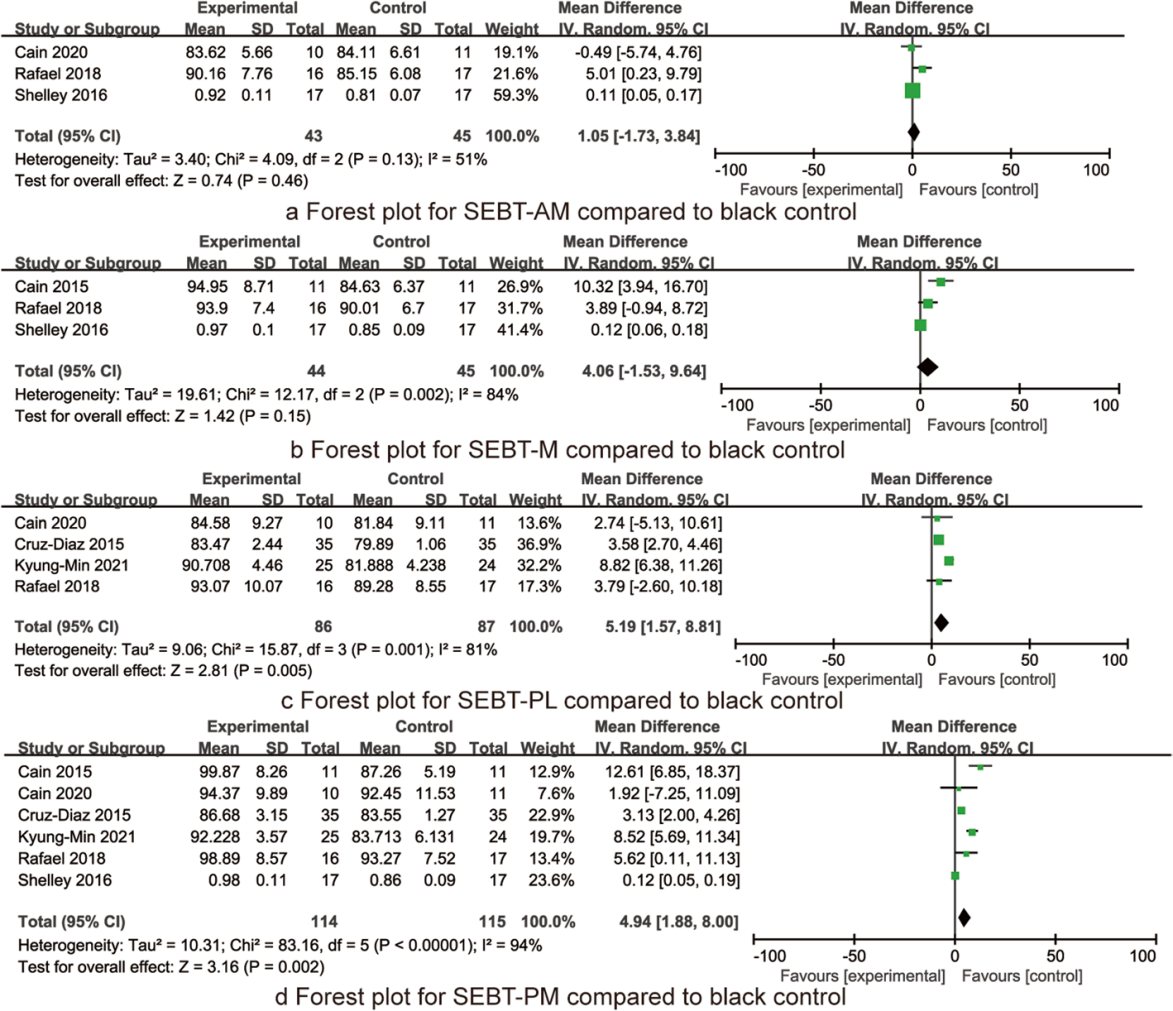
The forest plots for SEBT-AM, SEBT-M, SEBT-PL, and SEBT-PM compared to black control

Three RCTs reported the SEBT-AM after the intervention, including 88 patients. There was heterogeneity (*I*^2^ = 51%, *P* < 0.001), and the random effect model was used. Meta-analysis results showed that there was no significant difference in the SEBT-AM score between the balance training group and the blank control group [MD = 1.05, 95% CI (− 1.73, 3.84), *P* = 0.46, Fig. 3a].

Three RCTs reported the SEBT-M after the intervention, including 89 patients. There was heterogeneity (*I*^2^ = 84%, *P* = 0.002), and the random effect model was used. Meta-analysis results showed that there was no significant difference in the SEBT-M score between the balance training group and the blank control group [MD = 4.06, 95% CI(− 1.53, 9.64), *P* = 0.15, Fig. 3b].

Four RCTs reported the SEBT-PL after the intervention, including 173 patients. There was homogeneity (*I*^2^ = 81%, *P* = 0.001), and the random effect model was used. Meta-analysis results showed that compared with the blank control group, the SEBT-PL score of the balance training group was significantly improved [MD = 5.19, 95% CI (1.57, 8.81), *P* = 0.005, Fig. 3c].

Six RCTs reported the SEBT-PM after the intervention, including 229 patients. There was heterogeneity (*I*^2^ = 94%, *P* < 0.001), and the random effect model was used. Meta-analysis results showed that compared with the blank control group, the SEBT-PM score of the balance training group was significantly improved [MD = 4.94, 95% CI(1.88, 8.00), *P* = 0.002, Fig. 3d].

**Fig. 4:**
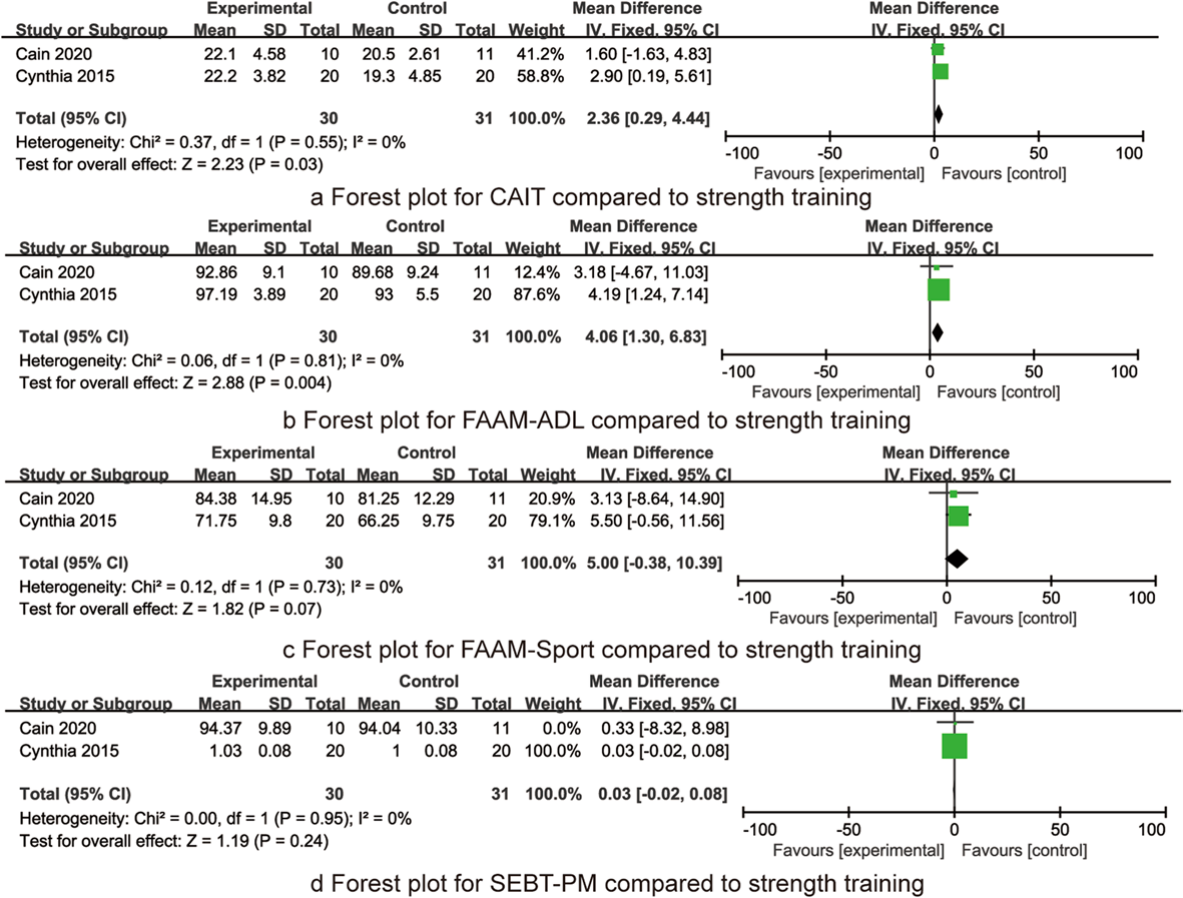
The forest plots for CAIT, FAAM-ADL, FAAM-Sports, and SEBT-PM compared to strength training

Two RCTs reported the CAIT after the intervention between the balance training group and the strength training group, including 61 patients. There was homogeneity (*I*^2^ = 0%, *P* = 0.55), and the fixed effect model was used. Meta-analysis results showed that compared with the strength training group, the CAI score of the balance training group was significantly improved [MD = 2.36, 95% CI (0.29, 4.44), *P* = 0.03, Fig. 4a].

Two RCTs reported the FAAM-ADL after the intervention between the balance training group and the strength training group, including 61 patients. There was homogeneity (*I*^2^ = 0%, *P* = 0.81), and the fixed effect model was used. Meta-analysis results showed that compared with the strength training group, the FAAM-ADL score of the balance training group was significantly improved. [MD = 4.06, 95% CI (1.30, 6.83), *P* = 0.004, Fig. 4b].

Two RCTs reported the FAAM-Sports after the intervention between balance the training group and the strength training group, including 61 patients. There was homogeneity (*I*^2^ = 0%, *P* = 0.73), and the fixed effect model was used. Meta-analysis results showed that there was no significant difference in the FAAM-Sports score between the balance training group and the strength training group [MD = 5.00, 95% CI (− 0.38, 10.39), *P* = 0.07, Fig. 4c].

Two RCTs reported the SEBT-PM after the intervention between the balance training group and the strength training group, including 61 patients. There was homogeneity (*I*^2^ = 0%, *P* = 0.95), and the fixed effect model was used. Meta-analysis results showed that there was no significant difference in the SEBT-PM score between the balance training group and the strength training group [MD = 0.03, 95% CI (− 0.02, 0.08), *P* = 0.24, Fig. 4d].

We conducted a subgroup analysis based on the duration and frequency and intensity of balance training to evaluate the homogeneity, the synthesized outcomes did not change statistically (all *P* > 0.05).

### Publication bias

Limited by as number of included studies, we could not perform a funnel plot. Regression analyses on the synthesized outcomes indicated that there was no publication bias (all *P* > 0.05).

### Sensitivity analysis

We systematically excluded RCTs for each individual result to assess whether the overall outcomes were affected. Our investigation revealed that the overall results remained unchanged regardless of excluding any specific RCT.

## Discussions

Balance is the ability to maintain a stable state of the body by resisting forces that interfere with the body [28]. The performance of impaired postural control and decreased postural stability in CAI patients may be caused by proprioception loss or neuromuscular control deficiency [29–31]. Therefore, improving the balance function of CAI patients can improve the functional activity of the affected ankle and reduce the risk of sprain again [32, 33]. The results of this analysis have shown that balance training improves daily activity ability, motor function, and dynamic balance to varying degrees. At the same time, comparing the results of balance training and strength training shows that there is no significant difference between the two training methods in improving the dynamic stability of CAI patients, while balance training has a more significant effect in improving the score of the functional scale.

CAIT is a scale to evaluate the existence and severity of ankle instability [34, 35]. Our results have shown that the stability of self-perception of CAI patients is improved after balance training. There is no significant heterogeneity between the research results of the CAIT questionnaire, but due to the insufficient number of included studies, more high-quality studies are needed. Activities of daily living and motor function can effectively reflect the activity and participation ability of patients [36–38]. FAAM is a very widely used scale to evaluate the functional activity ability caused by ankle joint-related injuries of lower limbs, which includes two self-assessment scales for the activity function in daily life and during exercise [39, 40]. For these two indicators, this study included two reports. Our meta-analysis results have shown that, compared with the control group that does not receive any intervention, the FAAM-ADL scores of CAI patients in the balance training group do not show significant differences, but the ability of sports activities is significantly improved. This may be because the impact of CAI proprioception damage on daily life was less than that of sports activities. At the same time, the ceiling effect of FAAM-ADL may also be one of the reasons why this analysis failed to draw significant differences, and the insufficient number of included studies is also worth considering.

SEBT can quantitatively measure changes in dynamic posture control and balance stability and can be used as an effective tool to measure patients’ dynamic balance [41–43]. Dynamic balance refers to the ability of the center of gravity to maintain the stability and direction of the body and posture during physical activities [44]. In daily life and various sports activities, the human body must constantly change the support plane and constantly adjust the body posture to meet the needs of balance [45, 46]. In SEBT, there are 5 directions (A front lateral, AM front medial, M medial, PM rear medial, and PL rear lateral) for analysis and comparison [47]. For the star bias test, SEBT-A, SEBT-AM, and SEBT-M were included in 5, 3, and 3 studies respectively. The results showed that there was no significant difference in the improvement of the stability of dynamic shift balance in each direction of the front side of the CAI patients in the test group who used balance training as an intervention method. Considerable heterogeneity was observed among the studies, potentially stemming from inadequate sample sizes, inconsistent baselines, varying intervention methods, and other factors. The results also substantiated a significant enhancement in the effectiveness of balance training on these two sets of indicators in both groups.

The analysis results show that balance training has advantages over strength training in improving the CAI and FAAM-ADL scores of CAIT patients, while there is no significant difference in improving FAAM Ports. This may be because CAI patients cannot resume all physiological activities to perform difficult tasks such as sports, even though some studies have shown that balance training is better than strength training in this respect [48, 49]. In the eight directions of the star bias test SEBT, only PM was included in more than one report. The results have shown that there is no significant difference between balance training and strength training in improving the dynamic stability of patients, which may be associated with the inconsistent baseline level between studies.

Some limitations of this meta-analysis are worth considering. Firstly, the number of included RCTs is small, and the sample size of included experimental studies is small. Secondly, the patients’ baseline levels were inconsistent and the training methods and parameters were different. Finally, some studies have not clearly pointed out the specific method of random allocation, whether the allocation method is hidden, and the implementation of blind methods for study populations. The quality of research methods included in the literature is different, leading to greater heterogeneity between various studies, which ultimately leads to the reduction of the credibility of research results. Future studies should be designed with more specific types of balance or strength interventions to determine appropriate and personalized exercise types, long-term prognosis, and patient compliance. At the same time, a comparison of baseline levels among included patients and an expansion of sample size should be added to increase the reliability of research results.

## Conclusions

In conclusion, the results of this meta-analysis have shown that balance training is beneficial to improve the daily living and sports ability of CAI patients, as well as the dynamic stability of the ankle joint on the posterior side. However, there is still disunity in the training methods, intensity, frequency, and duration of balance training, and there is still no standardized and specific balance training program for CAI patients. The development of a more specific balance training program can be one of the future research directions. Besides, high-quality research with a larger sample size on these issues in the future is needed in the future.

## Data Availability

All data generated or analyzed during this study are included in this published article.

## Abbreviations

CAI: Chronic ankle instability
RCTs: Randomized controlled trials
MD: Mean difference
CI: Confidence interval
MeSH: Medical Subject Headings
PRISMA: Preferred reporting items for systematic reviews and meta-analyses
CAIT: Cumberland Ankle Instability Tool
FAAM-ADL: Foot and Ankle Ability Measure ADL
FAAM-Sports: Foot and Ankle Ability Measure Sports
SEBT: Star Excursion Balance Test
